# 

**Published:** 1879-10

**Authors:** 


*CONTENTS.*

| | PAGE |
|---|---|
| Running Up Stairs | 1 |
| Philosophy of the Nervous System | 2 |
| Idiots | 3 |
| Seasonable Items | 4 |
| Changing Clothing | 5 |
| Our School System | 5 |
| Public School Outrages | 7 |
| Hospitals | 9 |
| Family Reading | 10 |
| Summerings | 12 |
| Death | 14 |
| Poison | 15 |
| Eight Hours a Day | 15 |
| Servant Plague | 18 |
| How to Live Long | 19 |
| Health | 21 |
| About Bees | 22 |
| Borax for Colds | 23 |
| Nutritive Value of Wheat Meal | 23 |
| Theory of Dreams | 24 |
| Sea Sickness | 24 |
| The Kitchen as the Arbiter of Human Destiny | 25 |
| Baking Meats | 26 |
| Honest Boys Make Honest Men | 27 |
| Sir Humphry Davy's Choice | 27 |
| The Arab's Proof | 28 |
| Poetry | 28 |
| A Thought for Parents | 29 |
| Anal Itchings | 29 |
| Digestion | 30 |
| Cathartics | 30 |
| The Sovereigns of England | 31 |
| Dr. Hall's Remedies | 32 |

